# Design and Expert-Based Usability Evaluation of a Smart Mobile Application for Aphasia Rehabilitation

**DOI:** 10.3390/healthcare14193273

**Published:** 2026-10-02

**Authors:** İbrahim Can Yaşa, Ayça Ak, Sezen Bal, Ayşe Yayla, Vedat Topuz, Fatih Kazdal, Ipek Midi, Zehra Yılmaz Eksen, Mehmet Can Koray Çekim

**Affiliations:** 1 Speech and Language Therapy Department, Faculty of Health Sciences, Bahçeşehir University, Istanbul 34353, Turkey; 2 Electronics and Automation Department, Vocational School of Technical Sciences, Marmara University, Istanbul 34865, Turkey; aycaak@marmara.edu.tr (A.A.); acetinkaya@marmara.edu.tr (A.Y.); vtopuz@marmara.edu.tr (V.T.); fatih.kazdal@marmara.edu.tr (F.K.); 3 Computer Technology Department, Vocational School of Technical Sciences, Marmara University, Istanbul 34865, Turkey; sezen.bal@marmara.edu.tr (S.B.); mcankoray@hotmail.com (M.C.K.Ç.); 4 Department of Neurology, Faculty of Medicine, Marmara University, Istanbul 34899, Turkey; ipekmidi@gmail.com; 5 Department of Speech and Language Therapy, Fenerbahce University, Istanbul 34758, Turkey; zylm@yahoo.com

**Keywords:** aphasia AI-assisted rehabilitation, mobile application, speech and language therapy, usability

## Abstract

Background/Objectives: This paper aims to develop an artificial intelligence-assisted mobile application using speech recognition and image processing technologies to address language difficulties experienced by individuals with aphasia. Methods: The mobile application was developed in the Android Studio environment using the Java programming language. Google Speech API was used for speech recognition, and Google ML Kit’s image labelling APIs were used for image recognition. The development process of the application was conducted using the Scrum methodology. Features developed during the sprints for each module were evaluated through Expectation Tests conducted with expert participation. In these tests, users’ pre-application expectations and post-application experiences with the modules were analysed through comparison. At the end of the development process, a System Usability Scale (SUS) Test was conducted to assess the overall integration of all modules. Results: Based on the results of Expectation Tests, necessary updates were made, and improvements were implemented in the functionality of each module. According to the SUS Test results, the application demonstrated an acceptable level of usability rate, and positive feedback was received in terms of user experience. The expert evaluation indicated that the application has acceptable usability and shows promise as a supportive tool for aphasia rehabilitation. Conclusions: This prototype application has the potential to support speech skills of individuals with aphasia, especially those who face challenges in accessing a Speech and Language Therapist. The integration of artificial intelligence-supported speech recognition and image processing technologies offers a promising supportive tool for the language rehabilitation of individuals with aphasia. Testing this prototype with a wider group of users and continuously updating it will be crucial in providing further support to individuals with aphasia.

## 1. Introduction

Aphasia is an acquired focal neurological disorder of language functions related to impairment in language production, comprehension, reading, writing, naming and repetition that develops due to damage to the brain areas responsible for language function. Aphasia is one of the most common cognitive-linguistic disorders after stroke. According to global reports from the World Stroke Organization, more than 12 million individuals worldwide experience a stroke each year, resulting in approximately 6.5 million fatalities. Consequently, an estimated one-third of stroke survivors—translating to over 4 million individuals globally—develop stroke-related aphasia annually [1]. Beyond language impairment itself, aphasia can substantially affect daily functioning and social participation. Individuals with stroke-related aphasia have been reported to experience lower quality of life and reduced participation in social activities compared with individuals without aphasia [2]. Difficulties in communication may also influence interpersonal relationships, autonomy, emotional well-being, and participation in daily activities. Reported psychological and social consequences include anxiety, fear, embarrassment, isolation, withdrawal, reduced confidence, and loneliness [3]. Depression has also been reported among individuals who develop aphasia following stroke, with one study reporting depressive symptoms in 60% of individuals at the end of the first year after stroke [4]. The importance of addressing aphasia after stroke is also reflected in research priorities: in a survey involving healthcare professionals and family members, aphasia treatment was ranked among the top priorities for stroke research, placing third among the ten identified priorities [5]. These consequences highlight the importance of accessible and sustained rehabilitation approaches that support the individual’s remaining communication abilities and reduce dependence on others.

There are studies showing that speech and language therapy is almost one of the main solutions in the treatment of aphasia and is effective in improving language disorders [6,7]. Evidence from aphasia rehabilitation studies suggests a dose–response relationship, whereby more intensive speech and language therapy may lead to greater language and communication gains, whereas lower therapy doses may provide more limited benefits [8,9]. Studies that offer less therapy are known to provide limited benefit [9]. In a meta-analysis study conducted by Bhogal et al. in 2003, it was reported that more positive results were obtained in groups with intensive therapy [10]. According to Basso’s study in 2005, intensive therapy for individuals with aphasia has a great effect on the development of language skills [11]. Therefore, more intensive therapy may be preferable when clinically feasible. When the frequency and duration of the therapy offered to individuals with aphasia are examined, it is recommended that aphasia therapy should be provided five days a week or at an intensity that the individual with aphasia can tolerate [12]. However, maintaining frequent and intensive face-to-face therapy may be challenging because of limitations related to access to specialized professionals, time, geographical distance, and opportunities for practice outside clinical settings. This creates a need for complementary rehabilitation approaches that can extend language practice beyond conventional therapy sessions.

The widespread availability of smartphones and tablets has created opportunities for delivering rehabilitation activities beyond conventional clinical environments. Mobile health applications can provide structured exercises, repeated practice, immediate interaction, and home-based access to therapeutic activities. In aphasia rehabilitation, these characteristics are particularly relevant because language recovery may require repeated and sustained practice. Mobile-based interventions can therefore serve as a complementary tool to face-to-face speech and language therapy by increasing opportunities for practice while reducing some accessibility barriers.

Previous studies have investigated the use of mobile and tablet-based applications in aphasia rehabilitation [13,14]. For example, Routhier et al. investigated the use of a tablet-based application for individuals with aphasia and reported positive outcomes in language-related abilities as well as favorable user experiences [14]. Similarly, Garcia developed a game-based application intended to support speech therapy and examined its usability with individuals with aphasia [15]. These studies indicate the potential of mobile technologies to support language rehabilitation; however, the design of an effective mobile rehabilitation application requires consideration of the diverse language and cognitive difficulties experienced by individuals with aphasia. Activities involving naming, repetition, sentence construction, auditory comprehension, and other language functions are commonly incorporated into speech and language therapy [16]. Different therapeutic approaches, including pragmatic, cognitive, functional, production and lexical, computer-assisted, semantic, social, and outcome-based approaches, have also been described [16].

Despite the potential of mobile technologies to extend aphasia rehabilitation beyond conventional clinical settings, the design of such applications should take into account the specific communication difficulties associated with aphasia. Rather than functioning as general-purpose language-learning tools, aphasia-oriented applications should provide accessible and structured activities that address multiple language and cognitive functions. In this context, the present study focuses on the development of an AI-assisted mobile application for aphasia rehabilitation that integrates speech recognition and image-processing technologies. The application was designed to support five complementary skill areas: Listening and Comprehension, Speaking, Writing, Visual Comprehension, and Memory. These components provide different forms of interaction and practice while addressing language-related abilities that may be affected following stroke.

In addition to the functional development of a rehabilitation application, usability is an essential consideration for its practical adoption in clinical and home-based settings. An application may provide technically functional exercises but still be difficult to use if its interface, instructions, navigation, feedback, or interaction mechanisms are not appropriate for the intended users. Therefore, the evaluation of usability should be considered alongside the technical development of the application. In the present study, the developed application was evaluated from an expert perspective to examine the suitability of its design, interaction structure, and rehabilitation-oriented functions. This assessment provides a complementary perspective to the technical features of the system and helps identify aspects of the application that may require improvement before broader use.

The aim of this study is to design, implement, and evaluate MARfazi, an AI-assisted mobile application designed to support speech and language rehabilitation in individuals with aphasia. Rather than replacing face-to-face clinical therapy, MARfazi is designed as a complementary, home-based rehabilitation tool to facilitate sustained, structured language practice across multiple domains, including auditory comprehension, verbal production, writing, visual comprehension, and semantic naming. In addition to the development of the application, the study specifically aims to assess its clinical relevance and usability through an expert-based evaluation protocol. The evaluation combines iterative sprint-level Expectation Tests with the standardized SUS and involves an interdisciplinary panel of 20 domain experts. Importantly, this evaluation serves as a pre-clinical feasibility and functional verification phase conducted exclusively with professional experts; it does not evaluate direct usability or therapeutic efficacy within the target patient population.

While commercially available digital aphasia tools provide a range of structured language exercises; however, differences remain in the extent to which speech interaction, visual recognition, and multiple rehabilitation-oriented functions are integrated within a single mobile platform. MARfazi was designed to explore this integrated approach by combining speech recognition, image-based interaction, and multiple language-oriented modules within a single Android application.

The remainder of this manuscript is organized as follows: Section 2 details the Scrum development process, clinical design principles, and software architecture; Section 3 reports the findings from the expert expectation tests and the SUS assessment; Section 4 provides a critical discussion of the results, technical limitations, and clinical implications; and Section 5 concludes the paper with directions for future clinical validation studies.

## 2. Materials and Methods

**Figure 1:**
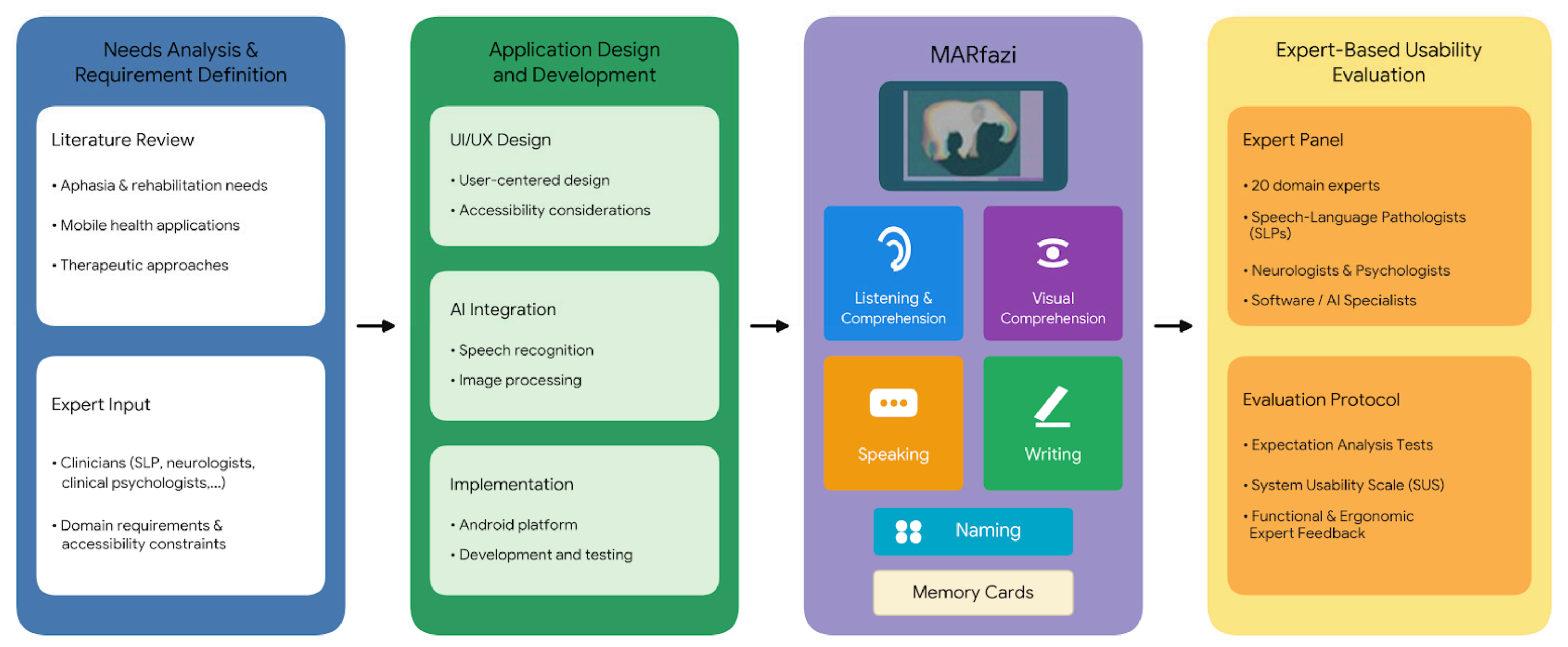
Comprehensive methodology of the system.

**Figure 2:**
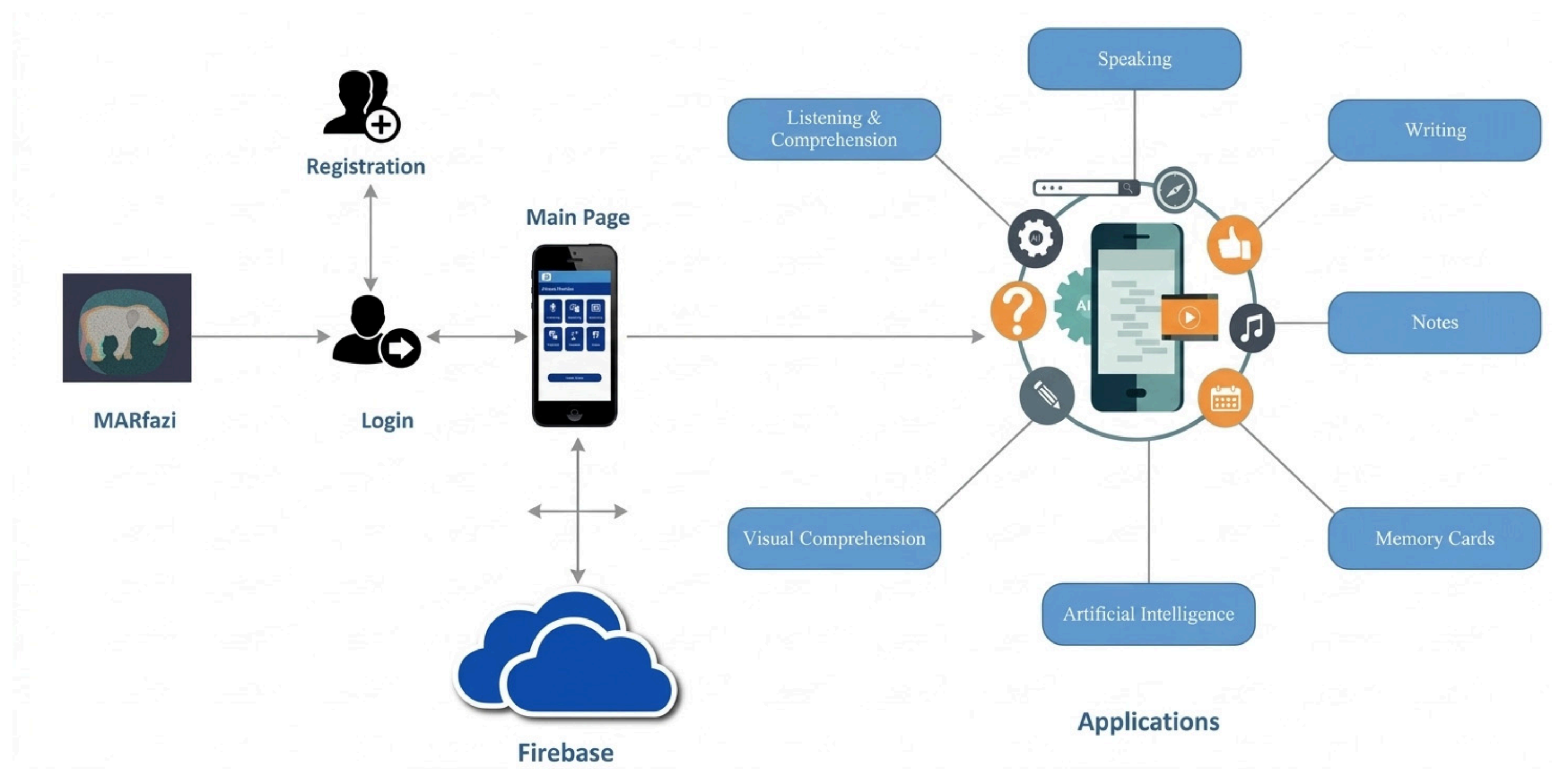
Block diagram of the system.

MARfazi is an Android-based mobile application designed to support individuals with aphasia in practicing reading, writing, comprehension, naming, and repetition skills. The comprehensive methodology is illustrated in Figure 1, while the overall software architecture and database connectivity are shown in Figure 2.

### 2.1. Development Process

The development process of the mobile application designed for aphasia patients was carried out using Scrum methodology. Scrum is a framework based on the principles of flexibility and continuous improvement in software development and project management [17]. This structure enables the project to be managed more effectively through specific roles, meetings and processes. There are three main roles in Scrum: Product Owner, Scrum Master and Development Team. Each takes on different but critical responsibilities in the progress of the project. The project team is organized in an interdisciplinary structure consisting of professionals from different fields of expertise such as neurologists, speech and language therapists, and academics. The Product Owner is an experienced expert in the field of speech and language therapy. Serving as a vital bridge between therapy practice and technical design, this expert identifies features that reflect clinical effectiveness and patient needs by gathering continuous feedback from speech therapists and clinicians. The Scrum Master role is taken by an academic who ensures that the project proceeds in accordance with Scrum principles and organizes communication within the team. The Development Team consists of academics and software developers who manage the technical development.

The sprints in the project were organised in time frames, usually lasting three weeks. At the beginning of each sprint, the Product Owner identified the features selected from the Product Catalogue and planned to be implemented during the sprint. The Development Team added tasks to the Sprint Catalogue to implement these features. Each day during the sprint, short Daily Scrum Meetings were organised where team members shared their daily progress and any problems they encountered.

Two important meetings were held at the end of each sprint: Sprint Review and Sprint Retrospective. In the Sprint Review meeting, the features worked on by the development team during the sprint were demoed and evaluated by the Product Owner and other clinical experts. This evaluation allowed the application to be examined in terms of interface usability, functional suitability, and clinical relevance. The Sprint Retrospective meeting aimed to improve the process and collaboration. The team reviewed the challenges and successes encountered throughout the sprint and suggested improvements for future sprints.

Expectation tests were conducted at the beginning and end of each sprint. These tests were conducted before and at the end of the sprint to assess how well the application met the criteria set by the experts.

### 2.2. Clinical Design Principles

The clinical design of MARfazi was guided by an interdisciplinary team consisting of speech and language therapists, neurologists, and software developers throughout the development process. The primary objective was to create a mobile application that supports commonly targeted language domains in aphasia rehabilitation while maintaining a simple and intuitive user interface.

The application modules were designed according to fundamental language rehabilitation principles commonly used in speech and language therapy. Rather than replacing conventional therapy, the application was developed as a supportive home-based practice tool to reinforce therapeutic exercises. Accordingly, separate modules were implemented to target different communication domains, including auditory comprehension, speech production, writing, visual comprehension, naming, and memory support. This modular structure enables therapists and users to focus on specific language functions according to individual rehabilitation needs.

The content of each module was determined through iterative discussions with clinical experts during Scrum review meetings. Speech and language therapists provided recommendations regarding the appropriateness of language tasks, instructions, and exercise content, while neurologists evaluated the overall suitability of the application for individuals with aphasia. Based on this expert feedback, revisions were incorporated into subsequent development iterations. The clinical targets of the MARfazi modules and the language functions they support are given in Table 1.

Accessibility and ease of use were considered throughout the interface design process. Simple navigation, large interactive elements, straightforward task flows, and immediate feedback mechanisms were implemented to reduce unnecessary cognitive load during application use. Audio instructions and visual supports were incorporated whenever appropriate to facilitate task completion. The expert-based usability evaluations conducted after each development iteration further guided interface refinements before the final System Usability Scale assessment.

The architecture of MARfazi is grounded in established speech-language therapy frameworks, primarily drawing on semantic feature analysis, lexical retrieval models, and functional/cognitive communication approaches [16]. Specifically, the AI-Assisted Object Recognition and Visual Comprehension modules incorporate semantic and cognitive principles to reinforce visual-lexical associations and stimulate naming pathways. The Speaking and Writing modules build upon lexical production models, encouraging structured expressive output via cued verbal and orthographic tasks. In parallel, the Listening & Comprehension, Memory Cards, and Notes modules leverage functional and pragmatic paradigms, fostering daily sentence processing, personal retention strategies, and practical communication independence. Table 1 maps each module to its foundational rehabilitation framework, targeted language functions, and clinical objectives.

### 2.3. Mobile Application

The application was developed utilizing Java and implemented within the Android Studio integrated development environment. In the process of creating the mobile application for aphasia rehabilitation, various modules were systematically developed across multiple development iterations. These modules include User Login, Database and Security, Listening and Comprehension, Speaking, Writing, Visual Comprehension, Artificial Intelligence (AI) Assisted Object Recognition, Memory Cards and Notes. Each module is designed to facilitate and support users’ language and communication skills through diverse methodologies. The Login module enables secure user authentication. The Database and Security module manages database operations, data encryption and protection against security vulnerabilities. Firebase was used as the database. Firebase is a real-time database that provides various services, including authentication, storage, hosting, and analytics.

The visual comprehension module is structured across multiple iterations to progressively increase complexity and is designed for individuals to develop this cognitive skill. Through the application, users can capture images and interact with AI-Assisted Object Recognition-powered object recognition technology that facilitates the coordination of visual and language processing abilities. Each activity within the application not only targets essential cognitive and language skills but is also structured to document the user’s progress, enabling therapists and users to receive specific feedback.

**Figure 3:**
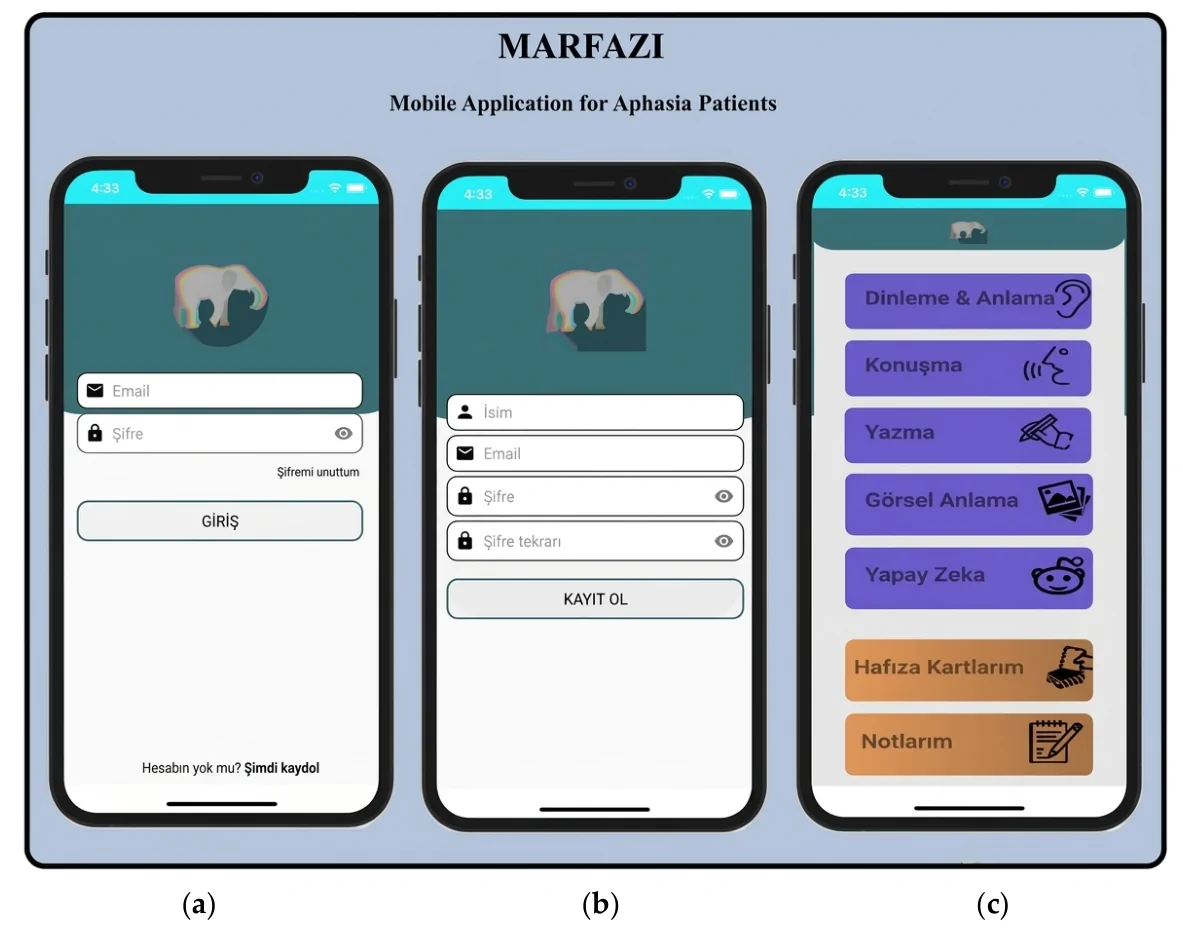
User registration, login and home page (Turkish locale): (**a**) Login Page; (**b**) First Registration; (**c**) Main Page. The Turkish interface labels correspond to Şifre (“Password”), Giriş (“Login”), Kayıt Ol (“Register”), Dinleme & Anlama (“Listening & Comprehension”), Konuşma (“Speaking”), Yazma (“Writing”), Görsel Anlama (“Visual Comprehension”), Yapay Zeka (“Artificial Intelligence”), Hafıza Kartlarım (“My Memory Cards”), and Notlarım (“My Notes”).

When the individual with aphasia (or his/her family) installs the application on his/her phone or tablet and runs it for the first time, the registration and login page appear (Figure 3a,b). The user is registered with Firebase by providing the necessary controls on the registration page. Following successfully logging in to the application, the user is then directed to the main page, which contains the following activities: Listening & Comprehension, Speaking, Writing, Visual Comprehension, AI-assisted object recognition, My Memory Cards, My Notes. Each module contains various applications designed to enhance the user’s speech and listening abilities (Figure 3c).

#### 2.3.1. Listening & Comprehension Module

**Figure 4:**
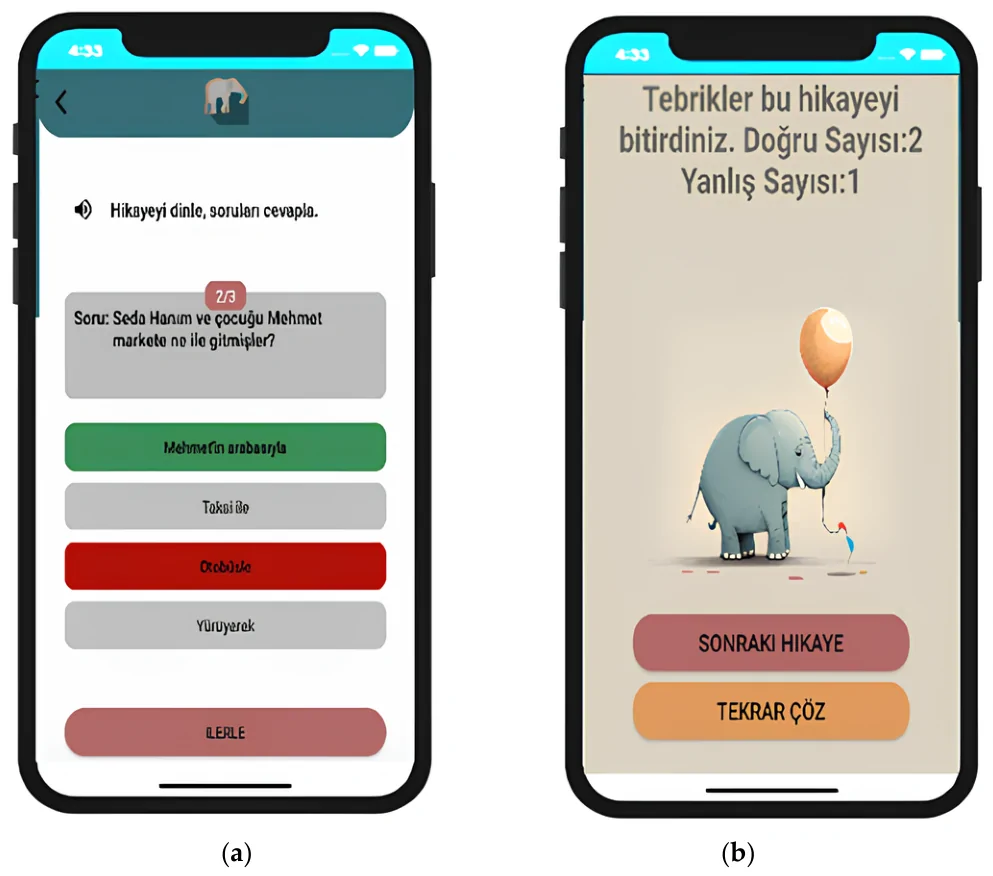
User interface of the Listening and Comprehension module. Showing (**a**) audio playback and question presentation, (**b**) immediate performance feedback to support self-guided practice, the Turkish interface labels correspond to Hikayeyi dinle, soruları cevapla (“Listen to the story and answer the questions”); Soru: Seda Hanım ve çocuğu Mehmet markete ne ile gittiler? (“Question: How did Ms. Seda and her son Mehmet go to the market?”); Mehmet’in arabasıyla (“By Mehmet’s car”), Taksi ile (“By taxi”), Otobüsle (“By bus”), Yürüyerek (“On foot”) and İLERLE (“NEXT”), Tebrikler bu hikayeyi bitirdiniz (“Congratulations, you have completed this story”), Doğru Sayısı: 2 (“Number of correct answers: 2”), Yanlış Sayısı: 1 (“Number of incorrect answers: 1”), SONRAKI HİKAYE (“NEXT STORY”) and TEKRAR ÇÖZ (“TRY AGAIN”).

Figure 4 shows the design of the Listening & Comprehension module. In this module, after the user selects the ‘Listening & Comprehension’ activity from the main page of the application, a story or audio recording from the Firebase database is automatically started. This story contains a dialogue or a short plot from daily life and is specially prepared to test users’ language comprehension abilities. After listening to the story, the user is asked a few questions related to the story. The questions are directly related to the content of the story and are designed to measure how well the user understands the story. For example, the user is asked questions such as ‘What did the main character in the story do?’ or ‘Where is the place in the story?’. The user responds by selecting the correct answer from the options on the screen. When the user answers the questions, the application provides instant feedback. If the answer is correct, the user is shown an encouraging message. If the answer is incorrect, they tick until they find the correct answer. This process is repeated to support the users’ learning process and improve their listening skills.

#### 2.3.2. Speaking Module

Speech recognition technology plays a critical role in the functionality of the application. This module uses Google’s Speech API which is based on automatic speech recognition technology. Automatic speech recognition has been the subject of extensive research. It encompasses the process of transforming spoken language into written text and finds applications across numerous domains, such as virtual assistants, transcription services, and tools for enhancing accessibility. The precision of automatic speech recognition systems has markedly enhanced due to advancements in deep learning methodologies. The Speech-to-Text service leverages Google Cloud’s foundational model, which is trained on millions of hours of audio data and billions of text sentences. This approach diverges from traditional speech recognition techniques that rely heavily on extensive amounts of language-specific supervised data. Such methods enhance recognition capabilities, accommodating a broader range of spoken languages and accents.

During this activity, participants select from categories including daily speech, animals, and objects, and words pertinent to the chosen category are presented. In the speaking phase, patients are expected to accurately pronounce the provided words. The primary objective of this activity is to foster the development of speaking skills.

#### 2.3.3. Writing Module

**Figure 5:**
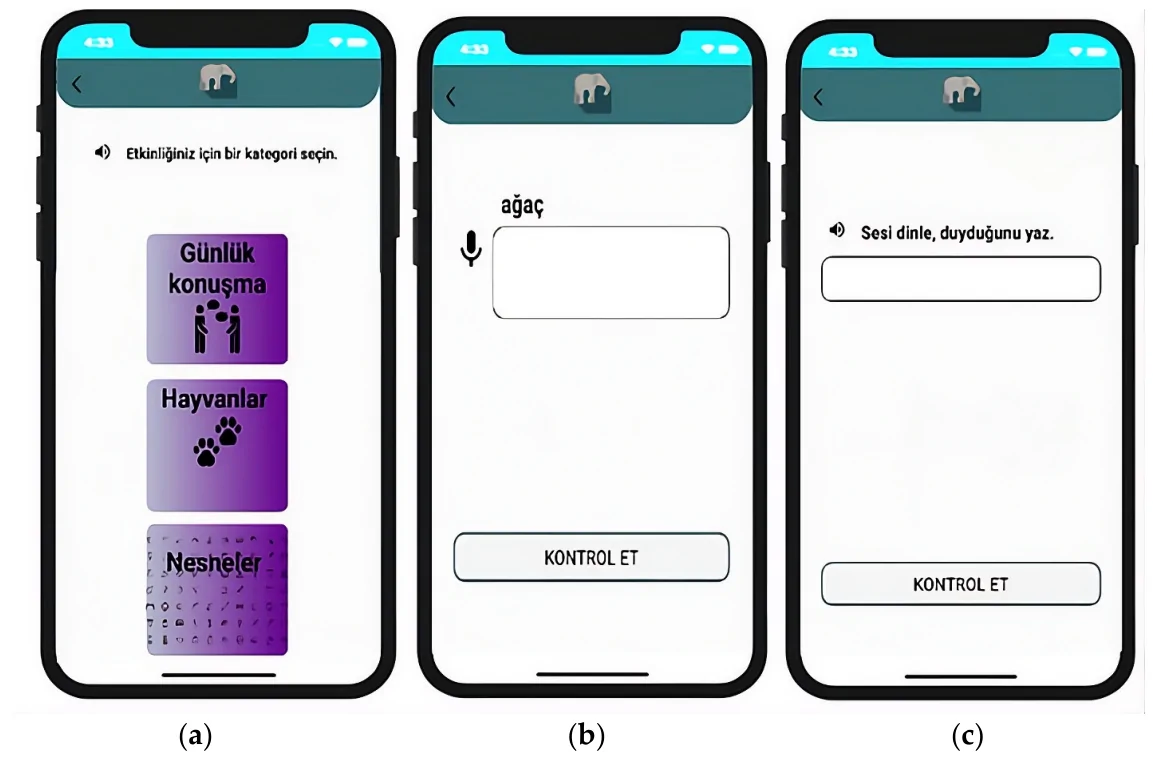
Speaking and writing module: (**a**) category selection, (**b**) speaking module with Speech API-based pronunciation checking, and (**c**) writing module combining auditory prompts with orthographic input. Turkish interface labels correspond to Etkinliğiniz için bir kategori seçin (“Select a category for your activity”), Günlük konuşma (“Daily conversation”), Hayvanlar (“Animals”), Nesneler (“Objects”), ağaç (“tree”), Sesi dinle, duyduğunu yaz (“Listen to the audio, write what you hear”) and KONTROL ET (“CHECK/VERIFY”).

In this activity, patients choose one of the categories such as daily speech, animals or objects. After the selection is made, a randomly selected utterance from the database of the selected category is read aloud. In the writing phase, patients are asked to listen carefully to the phrase and write it down correctly. When the typing is complete, the ‘Check’ button should be pressed. An auditory alert is heard when the correct answer is given. Figure 5 shows the visuals of the speaking (Figure 5a,b) and writing activity module (Figure 5c).

#### 2.3.4. Visual Comprehension Module

The Visual Comprehension Module aims to improve users’ visual perception skills at different levels. The module pulls content from a database containing images suitable for each difficulty level. Through the presented visuals, users’ recognition and comprehension skills are tested and improved through various activities.

Randomly selected images are presented to the user with the help of a specific algorithm from the visual data retrieved from the database. The user tries to recognize these images. Each correct recognition allows the user to move to the next level, while incorrect recognitions give the user the opportunity to try again. This process helps the user to gradually increase their visual perception skills.

**Figure 6:**
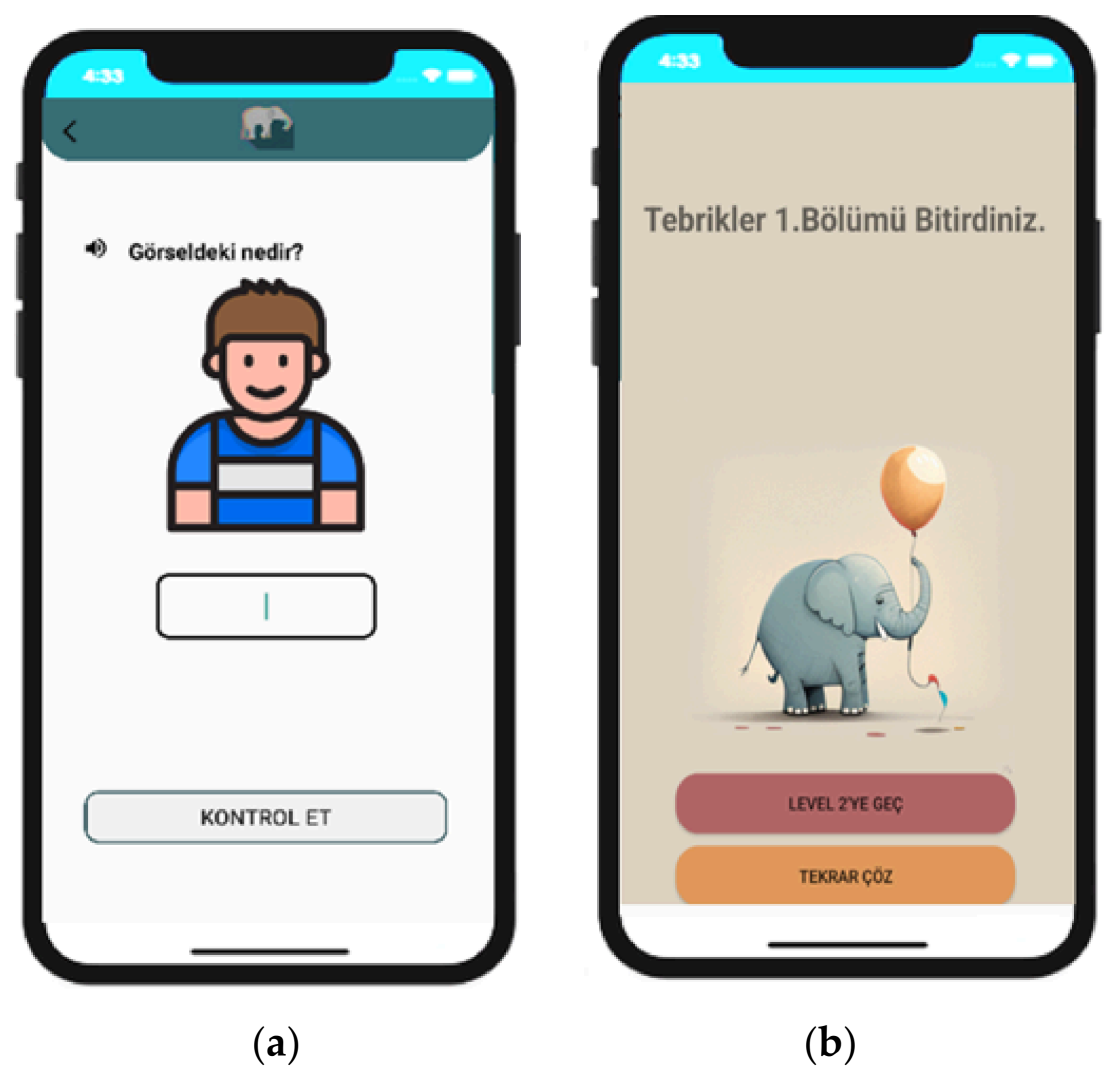
Visual Comprehension Module: (**a**) image recognition task across graded difficulty levels, and (**b**) completion screen providing progress tracking and reinforcement. he Turkish interface labels correspond to Görseldeki nedir? (“What is in the image?”), KONTROL ET (“CHECK/VERIFY”), Tebrikler 1. Bölümü Bitirdiniz. (“Congratulations, you have completed Chapter 1.”), LEVEL 2’YE GEÇ (“PROCEED TO LEVEL 2”), and TEKRAR ÇÖZ (“SOLVE AGAIN/RETRY”).

The module also monitors how accurately the user recognizes images and provides feedback accordingly. If the user recognizes an image correctly, they advance one level; if they get it wrong, the learning process is supported with feedback. This functionality allows the user to continuously learn and improve their skills. Figure 6 shows the screenshots of the visual comprehension activity.

#### 2.3.5. AI-Assisted Image Recognition

The AI-assisted object recognition module was developed to support object naming and visual-language association exercises. Rather than implementing a custom AI model, the application integrates Google’s ML Kit image labeling service to recognize objects captured by the device camera. This approach enables reliable on-device object detection while reducing computational requirements and simplifying deployment on Android devices. The recognized object labels are subsequently presented to the user as visual and textual cues to support naming practice.

After capturing an image using the device camera, the detected object label is displayed beneath the image and can also be played back as audio. The module is intended to reinforce object recognition, semantic association, and naming practice by providing immediate multimodal feedback. Users may store recognized objects for repeated practice or remove incorrectly recognized images. The purpose of this module is not to perform clinical diagnosis, but to provide an interactive exercise environment that complements conventional speech and language therapy.

**Figure 7:**
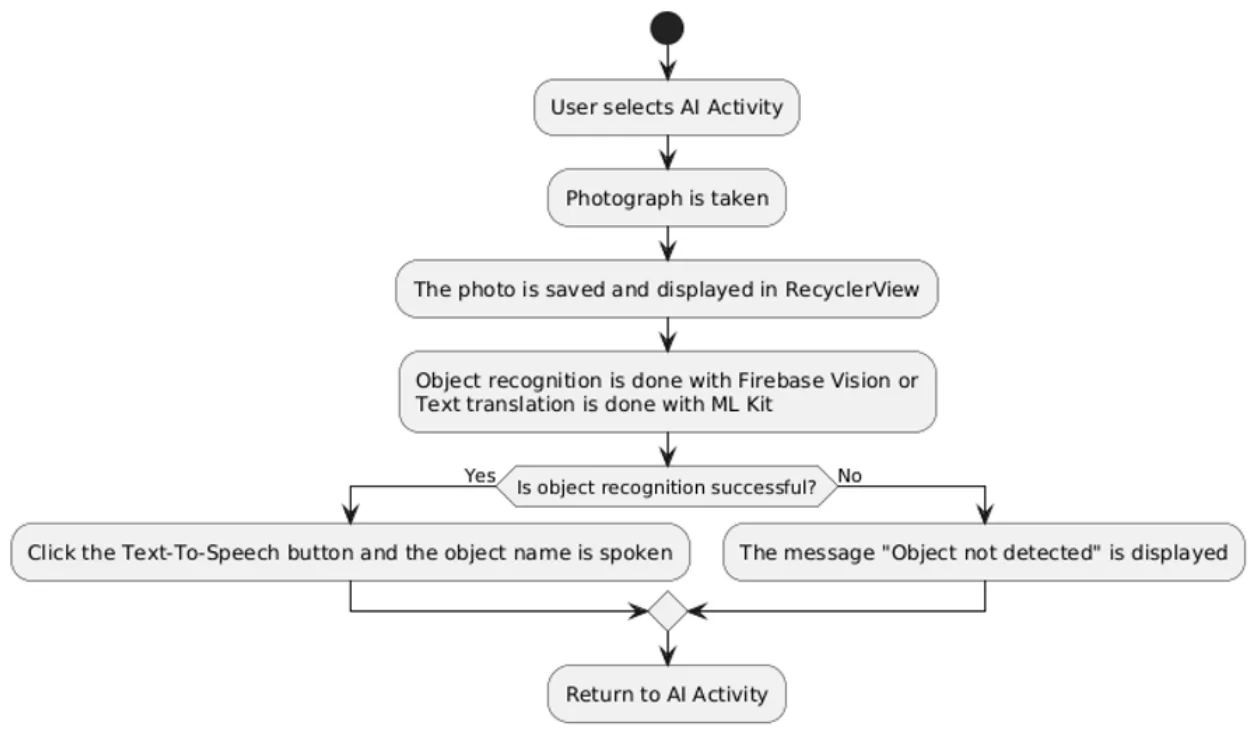
The flowchart of the AI-assisted object recognition module.

The speech recognition component was incorporated to provide immediate feedback during speaking exercises using a commercially available speech recognition service. Since the present study focused on application development and expert-based usability evaluation, the recognition accuracy for aphasic speech was not independently assessed. Future clinical studies involving individuals with aphasia will investigate the robustness of speech recognition under different speech impairment conditions and evaluate its impact on rehabilitation performance. The flowchart of the AI-Assisted Object Recognition Module is shown in Figure 7.

Because MARfazi leverages pre-trained mobile AI components, including Google ML Kit Image Labeling and the Android Speech Recognizer, through native API pipelines, the primary objective of this phase was the functional integration of these components into the application architecture rather than the development and benchmarking of custom neural network models.

#### 2.3.6. Memory Cards Module and Notes

The memory cards section was developed to help with image naming and memory problems. The user uploads a photo from his/her gallery or camera, gives it a name and saves it. The user can see the details of this saved memory card and can perform deletion operations. The user can do exercises with the saved cards from the exercise button. In the Note section, the user can save, delete, and update their notes.

This page allows users to turn on the camera and take pictures by clicking on the plus (+) sign in the upper right corner of the screen. The captured pictures are printed on the screen in cards and the object detection result is added under each picture. Users can listen to the audio pronunciation of the depicted object by tapping the ear icon in the upper-left corner of the card, or remove unwanted items using the delete icon in the upper-right corner.

### 2.4. Evaluation Protocol and Usability Assessment

The evaluation of the MARfazi prototype was conducted through an iterative User-Centered Design framework embedded within Agile-Scrum sprints. This methodology utilized a two-tier evaluation protocol: an iterative, formative Expectation Test to diagnose operational discrepancies across development sprints, followed by a summative, standardized SUS assessment upon final integration. The formative phase served as an internal functional verification to ensure baseline expectations were met, whereas the summative phase quantified global usability.

The expert panel was recruited using purposive sampling from academic institutions, neurology departments, and private speech therapy clinics. Clinical experts were required to hold an accredited professional degree in their respective discipline, have at least 3 years of direct clinical experience in post-stroke neurorehabilitation or aphasia management, and have familiarity with digital or augmentative tools. Technical experts were required to hold a degree in software engineering, computer science, or a related field and have at least 3 years of experience in mobile application development or user interface evaluation. The final multidisciplinary panel comprised 20 experts (*N* = 20), including 14 speech and language therapists (clinical experience: 3–6 years), 1 neurologist (12 years of experience), 1 clinical psychologist (8 years of experience), and 4 software engineers (professional experience: 3–5 years). The same panel of 20 experts (E1–E20) participated in both the formative Expectation Tests and the final summative SUS assessment, ensuring consistency of the expert panel across the two evaluation stages.

#### 2.4.1. Iterative Module-Level Expectation Tests

Expectation tests were used to determine whether the implemented features met the expectations of domain experts and to identify areas requiring improvement before the final usability evaluation. The SUS assessment was subsequently performed to evaluate the overall usability of the integrated application. The testing process and evaluation were carried out in four stages:

I. Application of Expectation Questionnaires: First, structured questionnaires were administered to assess domain experts’ baseline expectations regarding specific operational features across each module. Responses were recorded using a 5-point Likert scale ranging from 1 (“Strongly Disagree”) to 5 (“Strongly Agree”). Experts scored their anticipated system behavior across all predefined criteria prior to interacting with the application.

II. Application Use and Experience Questionnaire: In the second stage, the experts were given access to the relevant modules of the application and asked to use the modules. In this process, technical problems or difficulties related to the user experience were noted. Experts were asked to analyse each feature by experiencing the functionality and ease of use of the application module.

III. Actual Experience Scoring and Comparison: After the module usage experience was completed, the experts were asked to re-score the features they had previously rated for each module with the same scale (between 1 and 5). In this stage, the experts rated each feature of the module based on their actual usage experience.

IV. Comparison of Scores and Evaluation of Results: In the last stage, the pre-test expectation scores were compared with the post-test scores based on the usage experience.

The testing process and evaluation were conducted for each application module using a structured module-specific test scenario. Although Expectation Tests were conducted for all application modules during development, the Listening & Comprehension module is presented here as a representative example because the same assessment methodology was applied across all modules. A separate test scenario was defined for each module, and each scenario was implemented through a sequence of predefined test steps describing how the intended user interaction and system response were to be performed and evaluated. The Listening & Comprehension module was selected as the representative example because its workflow includes the main interaction stages evaluated in the Expectation Tests, including audio loading and playback, question presentation, answer processing, and feedback delivery. Presenting this module as a representative example allows the evaluation procedure to be described in detail without repeating similar test procedures for the other modules.

The test scenario for the Listening & Comprehension module was designed to evaluate the sequence of user interactions and corresponding system responses during an audio-based question-answer task. For the scenario to be implemented, it is a prerequisite that the user is logged in to the application. After logging in, the user can access all modules. In this context, the test steps for the Listening & Comprehension module are as follows:

- The user starts the Listening & Understanding module.
- The application presents an audio file to the user and provides a play button for the user to listen to the file.
- The user presses the play button and starts listening to the audio file.
- At the end of the audio file, the application automatically presents the user with a series of multiple-choice questions related to the module.
- The user answers each question and presses the ‘Submit Answers’ button to submit their answers.
- The application evaluates the user’s answers and provides feedback for each question, indicating whether it is correct or incorrect.
- The application provides a summary of the user’s overall performance and shows the correct answers.

#### 2.4.2. System Usability Scale (SUS) Protocol

Following the integration of all functional modules, an expert usability evaluation was conducted using the standardized 10-item SUS. SUS is a reliable and cost-effective survey method for assessing the usability of a product or service [19,20,21]. The scale contains ten statements, each of which questions a different aspect of the user experience and is divided into odd-numbered (positive) and even-numbered (negative) statements [22]. Respondents are asked to rate each statement from “strongly disagree” (1 point) to “strongly agree” (5 points). Individual item scores were converted according to standard SUS guidelines: for odd-numbered (positive) items, 1 point was subtracted from the user’s rating; for even-numbered (negative) items, the rating was subtracted from 5. The sum of the ten adjusted item scores was then multiplied by 2.5 to yield a normalized composite usability score on a scale from 0 to 100. A composite score of 68 was established as the benchmark threshold indicating acceptable usability. No individuals with aphasia participated in this preliminary testing phase. Consequently, the findings reflect expert-perceived usability, interface clarity, and functional suitability rather than user experience or therapeutic benefit experienced directly by individuals with aphasia.

## 3. Results

The findings of the expert-based evaluation are reported across two thematic sections: the sprint-level functional verification (Expectation Tests) and the final system-wide usability evaluation (SUS).

### 3.1. Expectation-Based Evaluation

During the iterative Scrum development cycles, expert Expectation Tests were administered across all modules to verify whether implemented features aligned with domain experts’ expectations. The Listening & Comprehension module serves as the representative implementation to illustrate this iterative evaluation process.

The distribution of expert ratings before use (expectations) and after hands-on interaction (actual experience) across five predefined criteria (C1: Audio Loading, C2: Audio Quality, C3: Question Presentation, C4: Answer Processing, and C5: Feedback Quality) is detailed in Table 2. Furthermore, Table 3 presents the individual score differentials (E = Experience-Expectation) recorded across all 20 participating experts.

As shown in Table 2 and Table 3, the vast majority of score differentials centered around zero, confirming a close correspondence between expert expectations and the actual functional performance of the application. Positive differentials were predominantly observed for criterion C4 and criterion C5, reflecting high satisfaction with task flow and immediate system responses. Conversely, slight negative differentials emerged in criterion C2 and criterion C5. Rather than indicating functional failure, these negative differentials served as direct pointers for software refinement, guiding improvements in audio clarity and instructional feedback in subsequent sprint iterations prior to final system integration.

To statistically substantiate whether expert expectations were met, a non-parametric Wilcoxon signed-rank test was conducted on paired pre-use expectation and post-use experience ratings across all five evaluation criteria (C1–C5). The analysis revealed no statistically significant divergence between anticipated and experienced scores for Audio File Loading (*Z* = −0.58, *p* = 0.56), Audio Quality (*Z* = −1.13, *p* = 0.26), Question Presentation (*Z* = −0.82, *p* = 0.41), Answer Processing (*Z* = −1.41, *p* = 0.16), or Feedback Quality (*Z* = −1.00, *p* = 0.32). The results showed no statistically significant differences between pre-use expectations and post-use experience ratings across the five criteria.

### 3.2. System Usability Scale Results

Following the integration of all functional modules and iterative refinements, the complete MARfazi application was evaluated using the standardized 10-item System Usability Scale (SUS) with the 20 domain experts. The individual responses to the ten SUS items and the calculated composite usability scores are presented in Table 4.

The overall mean SUS score obtained for MARfazi was 68.63 (±18.31), with individual scores ranging from 40.0 to 95.0. According to commonly used SUS benchmark guidelines, this score indicates an acceptable baseline level of perceived usability for the prototype. Ten of the 20 experts (50%) assigned SUS scores of 70.0 or higher, while five experts assigned scores of 87.5 or higher. The variability in individual scores indicates that perceptions of usability were not uniform across the expert panel. These lower ratings were primarily associated with expert observations regarding audio playback speed adjustment needs and the desire for more diverse visual reward mechanisms. This variability indicates that while the prototype achieves an acceptable operational baseline, further ergonomic optimization is necessary before clinical deployment with stroke survivors.

## 4. Discussion

The findings from the two evaluation stages provide complementary information about the developed application. The expectation-based evaluation identified specific discrepancies between anticipated and experienced functionality, offering practical feedback that guided iterative software refinements during the development sprints.

The subsequent SUS assessment provided a summative measure of perceived usability following system integration. In light of standardized usability benchmarks [21], the resulting average score suggests that the MARfazi prototype achieved an acceptable level of usability from an expert perspective. While the majority of domain experts awarded favorable ratings, the observed disparity between the highest and lowest individual evaluations indicates that multimodal mobile interfaces can introduce distinct usability challenges. This variability suggests that customizable interaction pacing and simplified navigation cues may warrant consideration in future patient-oriented evaluations.

The feedback gathered from our interdisciplinary panel corresponds with observations reported in earlier digital aphasia studies, where structured home exercises were suggested to support continuous language stimulation [13,14]. While prior work demonstrated positive user experiences through tablet-based activities [14,15], The application’s immediate visual and auditory feedback may facilitate task guidance during self-administered practice. In addition, utilizing on-device image labeling provides a practical way to connect real-world visual items with word-retrieval exercises, aligning with common naming objectives used in clinical practice [16].

Overall, the expert-based evaluations suggest that MARfazi successfully achieved its core architectural and usability objectives at the prototype level, serving as a functional feasibility baseline for home-oriented aphasia support. Within this developmental context, the primary scope of the present work focused on professional expert appraisal to ensure technical stability and therapeutic relevance prior to patient deployment. While direct trials with stroke survivors remain an essential subsequent phase, professional interdisciplinary feedback provided targeted benchmarks for interface ergonomics and cognitive pacing. Additionally, while standard speech recognition engines provide a practical entry point for automated voice feedback, future software iterations will focus on optimizing speech models. Consequently, this study provides an initial evidence base for further refinement and clinical validation, paving the way for prospective clinical trials to examine longitudinal compliance and therapeutic outcomes in real-world settings.

A critical technical limitation of the current prototype concerns its reliance on foundational, pre-trained on-device vision (Google ML Kit) and speech recognition (Google/Android Speech API) engines. While these native APIs support local processing with low perceived latency and reduce reliance on cloud-based processing, commercial automatic speech recognition pipelines are predominantly optimized for neurotypical acoustic profiles. Consequently, their baseline acoustic models often struggle with the phonetic distortions, dysarthric articulation breakdowns, prolonged pauses, and paraphasias characteristic of individuals with aphasia. Because Word Error Rates and classification accuracy were not independently benchmarked against disordered speech corpora or controlled image sets in this feasibility phase, the operational performance of the Speaking module remains contingent on standard acoustic assumptions. In clinical contexts, unrecognized speech or false-negative feedback could induce user frustration and directly hinder therapeutic engagement. Conducting structured bench tests alongside upcoming clinical trials, incorporating adaptive acoustic models, and establishing customizable confidence thresholds will be essential technical priorities to ensure the system robustly accommodates atypical speech patterns.

## 5. Conclusions

This study presented the design, implementation, and expert-based evaluation of MARfazi, an AI-assisted mobile application designed to provide multimodal support for aphasia rehabilitation. By integrating automated speech recognition and image-based interaction across structured language modules, the application provides a prototype platform for home-based language practice intended to complement conventional clinical therapy.

The iterative Scrum development process, expert Expectation Tests, and standardized SUS assessment provided preliminary evidence regarding the functional suitability and perceived usability of the prototype. However, because the evaluation involved domain experts rather than individuals with aphasia, the findings do not establish direct patient usability or therapeutic effectiveness. Future studies involving individuals with aphasia are therefore required to evaluate user experience, accessibility, adherence, and rehabilitation outcomes.

## Figures and Tables

**Table 1:** Relationship between modules, targeted language functions, and clinical objectives.

Module	Underlying Therapeutic Framework [16,18]	Targeted Language Function	Clinical Objective
Listening & Comprehension	Functional & Cognitive approaches	Auditory comprehension	Improve comprehension of spoken language through listening and question-answer tasks
Speaking	Lexical/Production & Computer-assisted approaches	Speech production, naming	Encourage verbal production and pronunciation practice
Writing	Orthographic & Lexical retrieval approaches	Written language	Improve spelling, word production, and sentence writing
Visual Comprehension	Cognitive & Semantic association approaches	Visual perception and semantic association	Strengthen visual recognition and language comprehension
AI-Assisted Object Recognition	Semantic feature & Multimodal naming approaches	Object naming and semantic retrieval	Support object identification and naming using AI-assisted image labelling
Memory Cards	Cognitive & Lexical reinforcement approaches	Memory and vocabulary reinforcement	Reinforce personalized vocabulary and recall
Notes	Functional & Pragmatic communication approaches	Functional communication	Support daily communication and personal reminders

**Table 2:** Listening & Comprehension Module Pre-Test Expectations and Post-Test Experience.

Criteria	Pre-Test Expectations		Post-Test Experience	
Audio File Loading	The audio file is expected to load quickly and seamlessly.	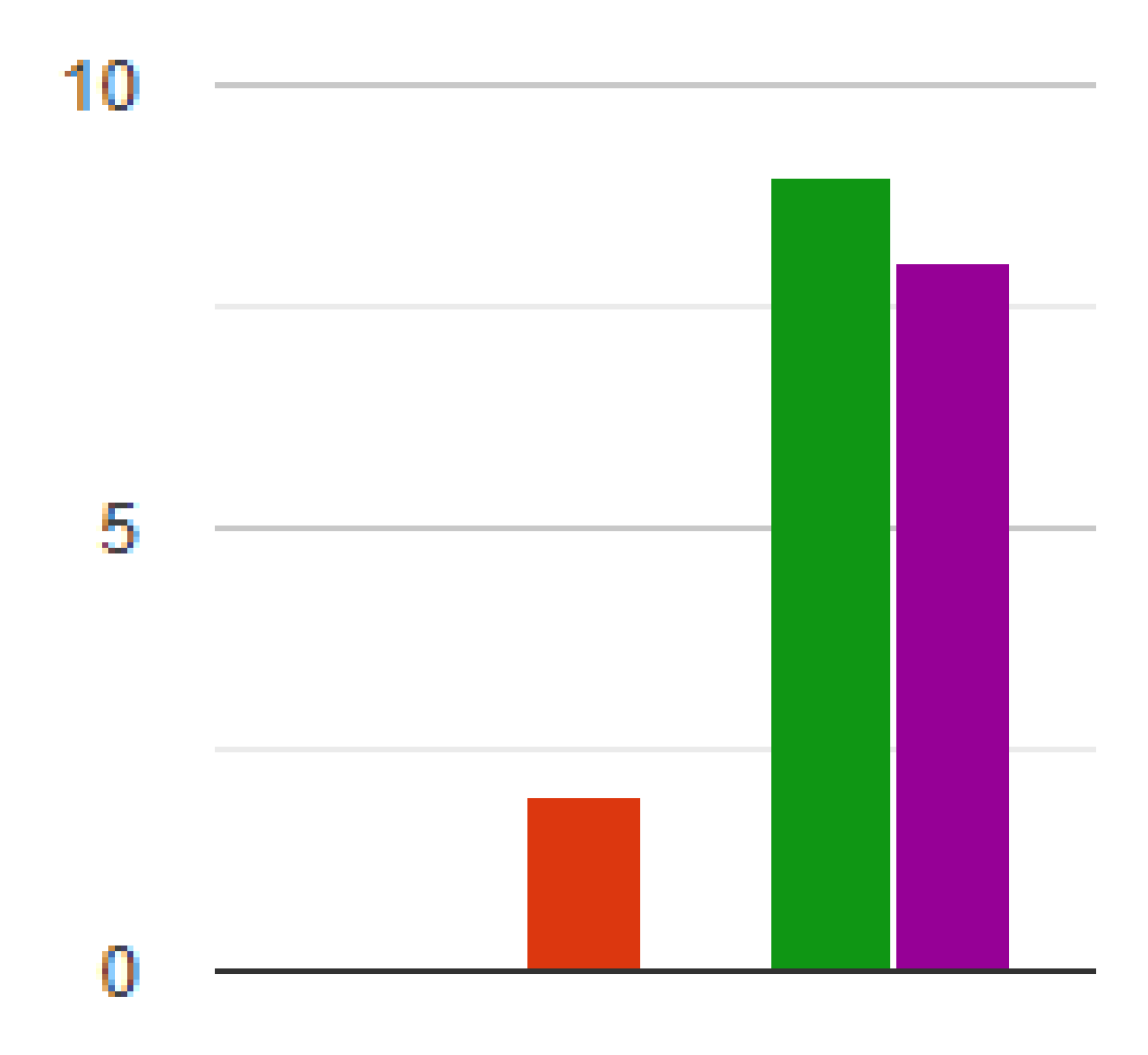	The audio file loaded quickly.	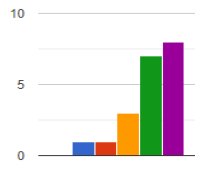
Audio Quality	Clear and understandable audio quality is expected.	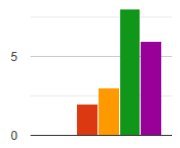	Audio quality was high.	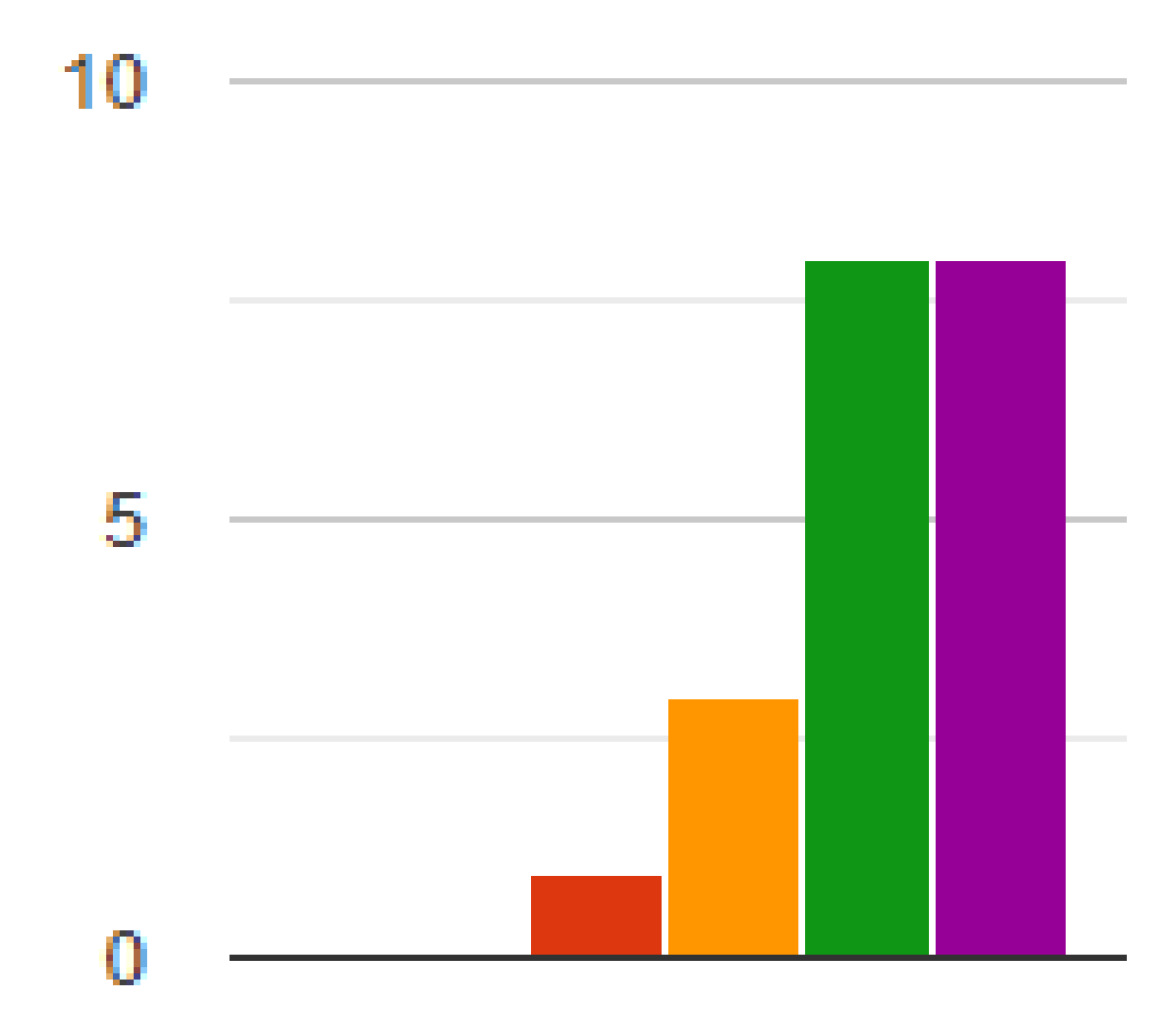
Question Presentation	Questions are expected to be clear and easily displayed in the user interface.	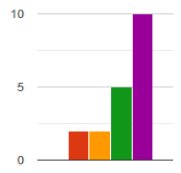	Questions were clear.	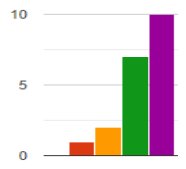
Answer Process	The user is expected to answer questions easily and the answers are processed quickly.	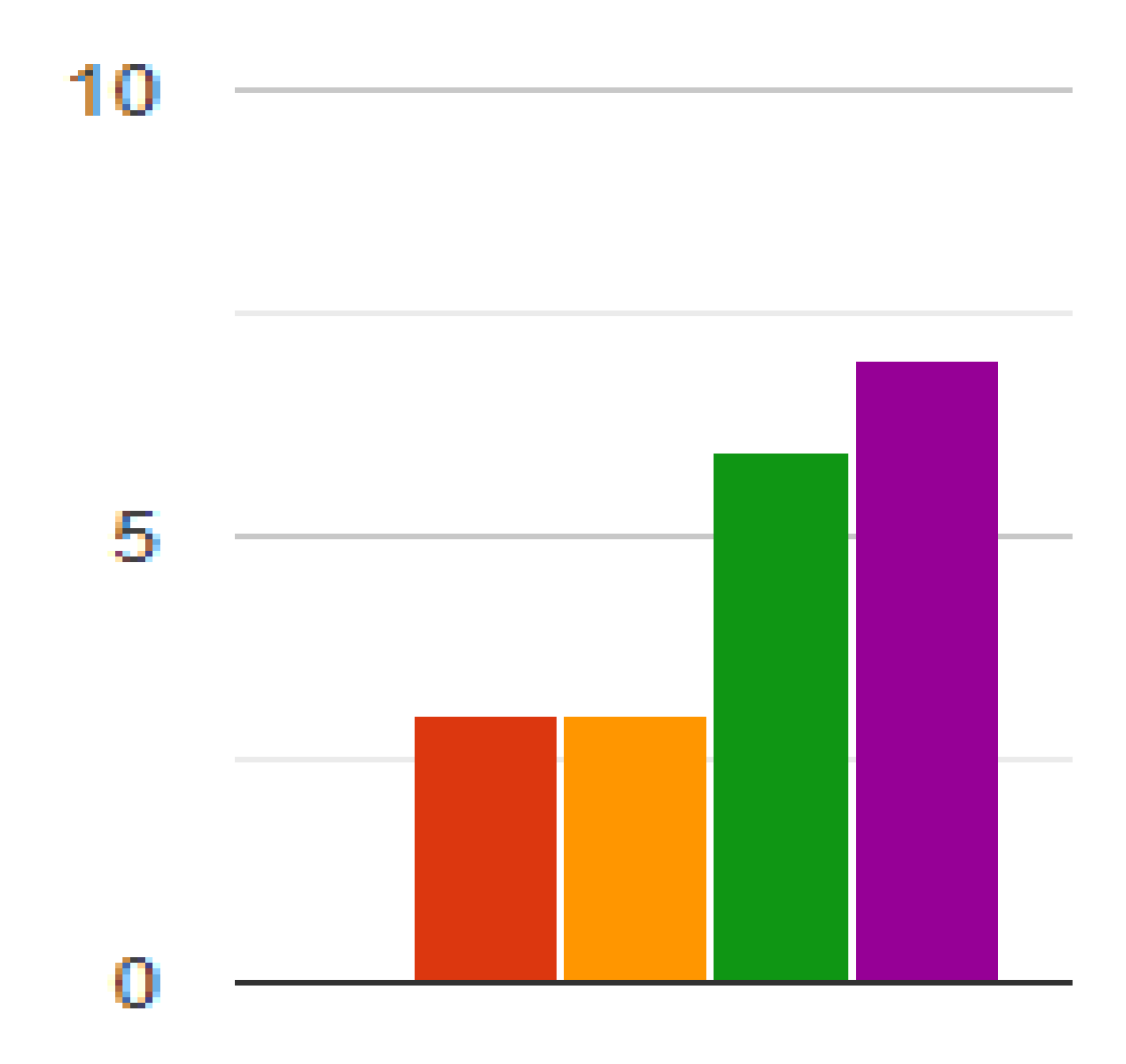	Answering process was easy.	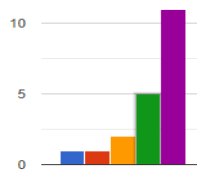
Feedback Quality	Clear and useful feedback based on the user’s answers is expected.	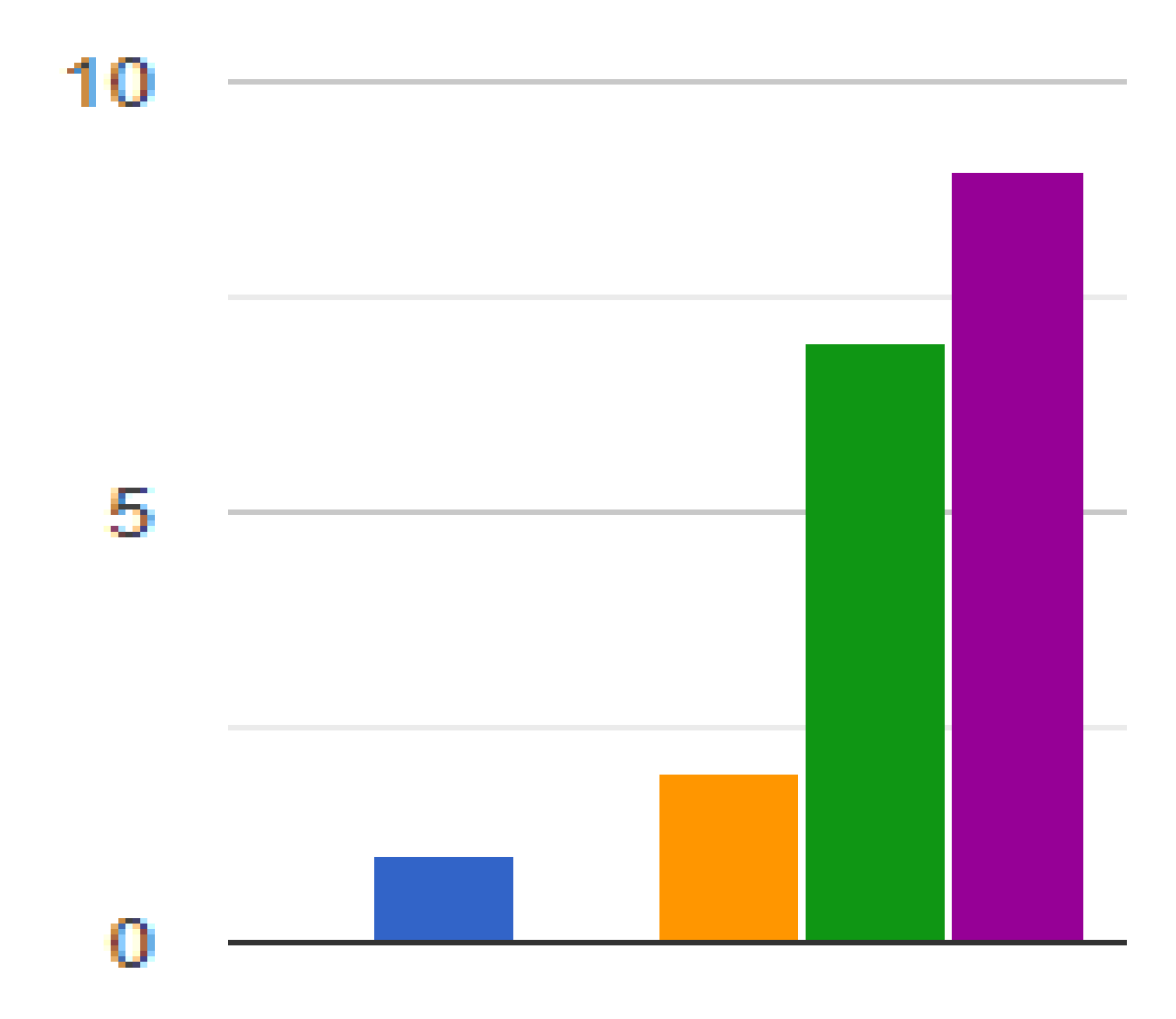	Feedback was useful.	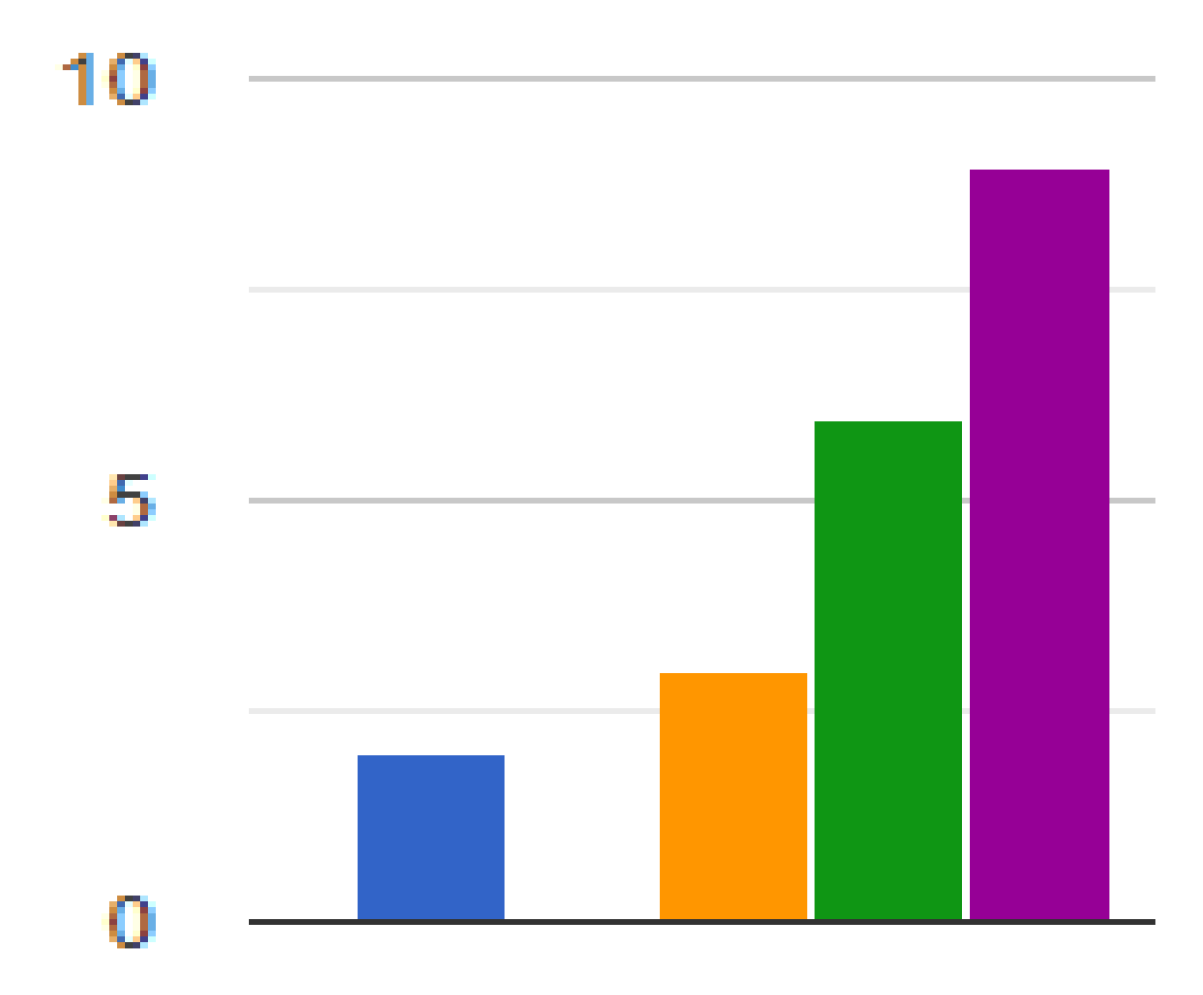

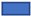
 1. Strongly disagree; 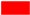 2. Disagree; 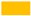 3. I am undecided; 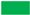 4. I agree; 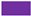 5. Strongly agree.

**Table 3:** Differences between Expectation and Experience for Listening & Comprehension Module.

	C1	C2	C3	C4	C5
E1	0	−1	−1	−1	−1
E2	0	0	0	0	+1
E3	0	0	−1	0	+1
E4	+1	+1	0	0	−1
E5	0	−1	0	+1	+2
E6	+1	0	+1	0	+1
E7	0	0	0	+1	+2
E8	0	+1	0	0	0
E9	−1	0	0	0	0
E10	+1	0	+1	0	0
E11	−1	0	−1	−1	0
E12	0	0	+1	−1	−1
E13	0	0	0	0	0
E14	0	0	0	0	0
E15	+1	0	0	0	0
E16	0	0	0	0	0
E17	0	0	0	0	0
E18	0	1	0	1	0
E19	0	0	0	0	0
E20	0	0	0	−1	0
Mean ± SD	0.05 ± 0.51	0.05 ± 0.51	−0.05 ± 0.60	0.00 ± 0.65	0.02 ± 0.77

Notes: E1–E20: Participating domain experts; C1: Audio File Loading; C2: Audio Quality; C3: Question Presentation; C4: Answer Processing; C5: Feedback Quality. Values represent score differentials. Pairwise comparisons via Wilcoxon signed-rank test indicated no significant divergence across criteria (*p* > 0.05 for all comparisons).

**Table 4:** Results of the SUS Test.

	Q1	Q2	Q3	Q4	Q5	Q6	Q7	Q8	Q9	Q10	SUS Score
E1	5	1	4	1	3	1	4	1	4	1	*87.5*
E2	5	2	4	5	5	1	1	5	2	2	*55*
E3	5	1	5	1	4	1	5	1	4	1	*95*
E4	4	3	3	2	4	1	2	4	1	3	*52.5*
E5	3	2	3	3	2	4	1	3	2	3	*40*
E6	3	2	3	2	4	2	4	1	3	2	*70*
E7	4	1	4	3	2	4	1	5	1	2	*42.5*
E8	3	1	4	3	1	1	5	1	4	1	*75*
E9	4	1	3	3	1	4	1	4	1	2	*40*
E10	3	1	4	3	1	1	2	1	2	1	*62.5*
E11	3	5	5	5	4	2	2	4	2	1	*47.5*
E12	4	3	4	1	1	4	3	1	4	1	*65*
E13	3	5	5	4	5	1	2	1	5	1	*70*
E14	4	1	5	5	4	1	2	5	5	1	*67.5*
E15	5	1	5	1	5	1	4	2	5	1	*95*
E16	5	4	5	2	5	1	5	1	5	1	*90*
E17	4	1	4	1	4	1	3	1	4	1	*85*
E18	3	2	4	3	4	3	4	3	3	2	*62.5*
E19	3	2	3	4	4	4	4	2	3	2	*82.5*
E20	5	2	4	1	4	2	5	1	4	1	*87.5*
	Average SUS Score										*68.63*

Notes: E1–E20: Participating domain experts; Q1–Q10: System Usability Scale items (scored 1 to 5); SUS Score: Normalized composite score (scale 0–100). Benchmark threshold for acceptable usability is 68.0.

## Data Availability

The datasets generated and/or analyzed during the current study are not publicly available because they contain participant-level evaluation data.

## Abbreviations

The following abbreviations are used in this manuscript:

AI	Artificial Intelligence
API	Application Programming Interface
SUS	System Usability Scale
ML	Machine Learning
